# Giant ovarian mucinous cystadenoma, a challenging situation in resource-limited countries

**DOI:** 10.1093/jscr/rjz366

**Published:** 2019-12-09

**Authors:** Miguel Angel Moyon, Daysi Alejandra León, William G Aguayo, Alejandro R Mecias, F Xavier Moyon, Jorge Tufiño, Alberto Yu, Gabriel Molina

**Affiliations:** 1 Attending Surgeon at IESS, HSFQ (Hospital General San Francisco), Department of General Surgery, Quito Ecuador; 2 PGY1 Surgery Resident at Pontificia Universidad Católica del Ecuador, Quito, Ecuador; 3 Attending Surgeon at IESS, Hospital Quito Sur, Department of General Surgery, Quito Ecuador

## Abstract

Mucinous cystadenomas are a common benign neoplasm of the ovaries that can grow much larger than other adnexal masses; they are recognized as precursors of ovarian cancer and may slowly transform to borderline tumors and invasive ovarian cancer. Prompt and accurate treatment is essential as these tumors can grow to massive sizes and be potentially lethal if left untreated. Health care providers must understand the patient, their expectations and their problems; without proper communication and follow-up, any treatment is destined to disappoint. We present a case of a 76-year-old female with limited access to health care. She presented with a giant cystadenoma that grew over 1 year. Complete resection was decided and the patient underwent complete recovery. On follow-up control patient is doing well.

## INTRODUCTION

Giant abdominal tumors are now rarely seen because of the improved imaging modalities and minimally invasive approaches, allowing the diagnosis to be made at an earlier stage. Adnexal masses can appear at any age of life and may be caused by both gynecologic and nongynecologic diseases. Imaging, lab markers and surgery are necessary to properly diagnose and treat these masses [1, 2, 3]. Cystadenomas are neoplasms lined by mucin-producing epithelial cells, and they are mostly benign; however, close follow-up is necessary as they are recognized as precursors of ovarian cancer and may slowly transform to borderline tumors and invasive ovarian cancer [1, 4]. These tumors are usually small; however, in rare scenarios especially in resource-limited countries where healthcare access is limited and patient noncompliance is expected, they can grow to massive sizes [1, 5]. We present a case of a 76-year-old female with a giant cystadenoma that grew over 1 year. Complete resection was decided and the patient underwent complete recovery.

## CASE REPORT

The patient is a 76-year-old female with a past medical history of diabetes and hypertension. She presented to our department with a 1-year history of a lower abdominal mass. She lived in a remote area so she did not have much access to healthcare. In the last 6 months, she noted that her abdomen was gradually distending until reaching enormous proportions. She remained completely asymptomatic and had no associated symptoms of constipation, vomiting or early satiety. She had delivered two children by vaginal delivery and they were all alive. Also, she did not smoke and did not drink alcohol or experience weight loss.

**Figure 1:**
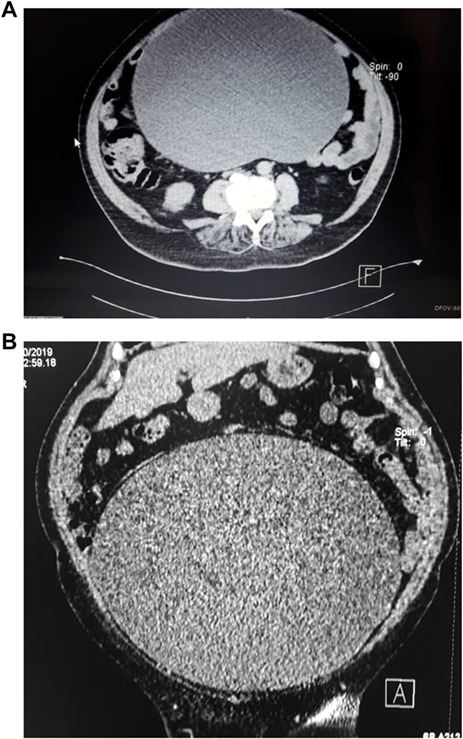
(A) CT scan, show giant cystic mass. (B) CT scan, showing ovarian tumor filling the lower abdomen.

**Figure 2:**
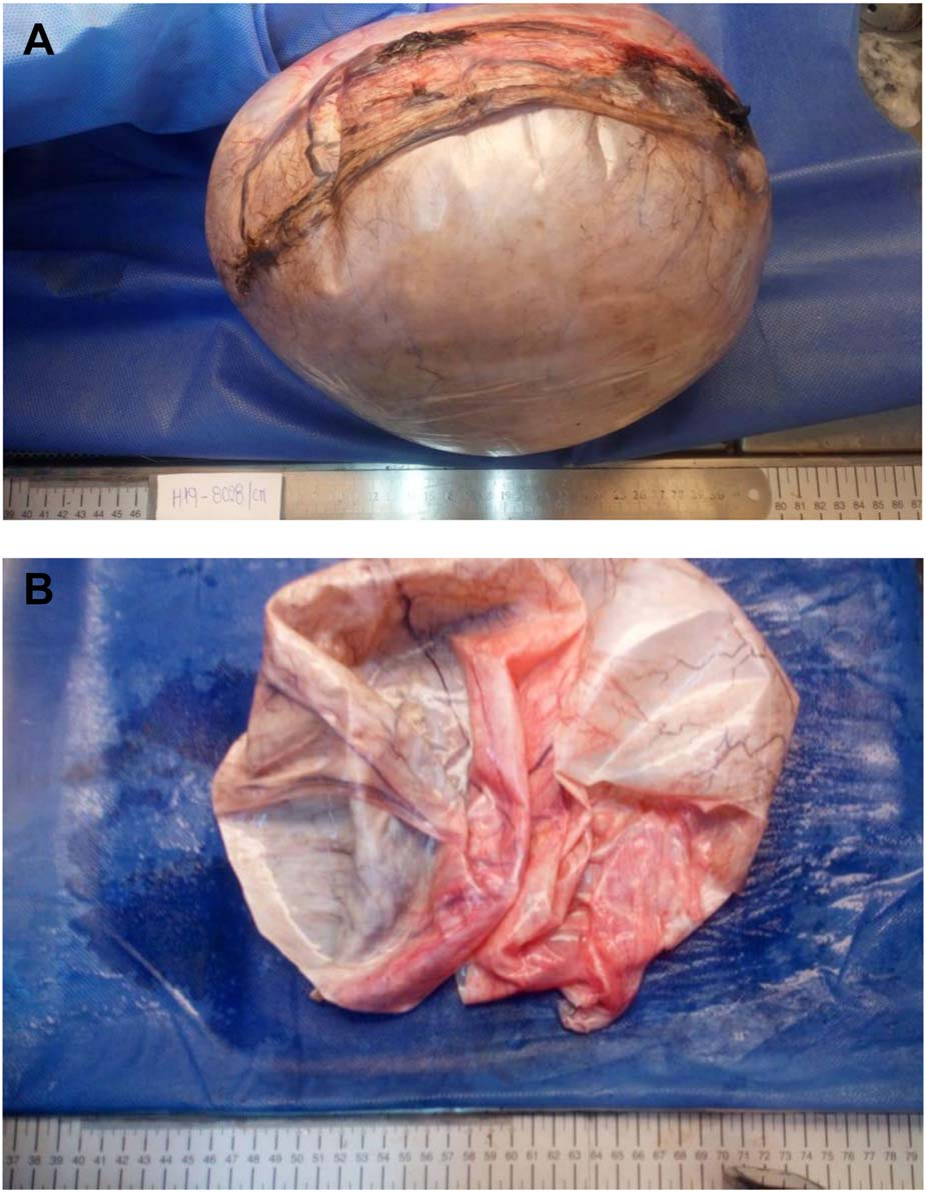
(A) Ovarian mass, its capsule had a whitish color and was covered with dilated blood vessels. (B) Ovarian mass, the tumor had 1000 cc clear fluid.

Upon examination, her abdomen was markedly distended; however, bowel sounds were normal and she did not have any abdominal discomfort. An abdominal echography revealed a giant heterogeneous mass with a thick wall and areas of internal septation. Due to this, an abdominal contrast-enhanced computed tomography (CT) was requested, revealing a giant 18.4 × 31.2 × 25.9 cm cystic lesion (4, 8 HU); the mass extended from the pelvis all the way through her upper abdomen, was dependent from the right adnexa and was in intimate contact with the bowel and omentum; however, the mass did not invade them. No lymph nodes or other masses were detected. (Fig. 1A and B) Complementary exams including CA-125, CAE and HE4 were normal. Due to the size of the mass, surgery was decided. On laparotomy, no ascites were identified; nonetheless, a 30 × 25 × 18 abdominopelvic mass was found filling most of her lower abdomen, and it was completely attached to the omentum and right adnexa. It had a whitish color and was covered with dilated blood vessels (Fig. 2A and B). Although there were no findings of lymph node metastasis or dissemination in the peritoneal cavity, we could not rule out that the tumor was solely benign, borderline malignant or malignant. Therefore, complete excision of the mass, along with complete omentectomy, appendectomy and close follow–up, was decided.

**Figure 3:**
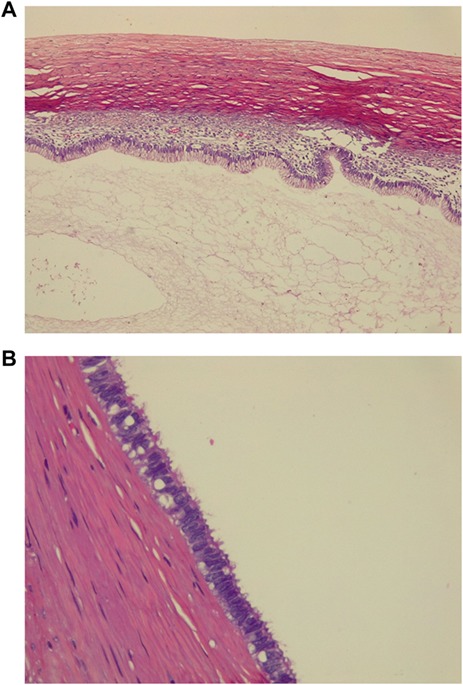
(A) Pathology, tumor is lined with nonciliated, mucin-secreting, columnar epithelium with goblet cells. (C) Pathology, ovarian mucinous cystadenoma without atypia.

Pathology revealed a 6 kg giant ovarian mucinous cystadenoma; its capsule measured 22 mm on average and was whitish with pink patches. Upon opening the cyst, 1000 cc of clear liquid was unveiled. The tumor was lined with nonciliated, mucin-secreting, columnar epithelium with goblet cells without atypia (Fig. 3A and B).

Her postoperative course was uneventful, sips of liquids were initiated on her third postoperative day and full diet was started soon after. She was discharged without complications. On follow–up, the control patient is doing well.

## DISCUSSION

Adnexal tumors are a common problem in postmenopausal woman, and due to its wide spectrum of clinical manifestations, they may present as an asymptomatic mass detected only as an incidental finding or as a giant abdominal tumor. Either one of these possibilities holds the likelihood of malignancy; nonetheless, due to the advancement at imaging technologies and health care services, ovarian tumors that reach these massive sizes are now rarely seen [1, 2]. In our patient, due to a lack of access to healthcare and the fact that she was completely asymptomatic, she allowed the mass to grow to gigantic proportions.

Ovarian tumors can be classified as stromal tumors (granulosa-theca, Sertoli and Leydig cells); germ cell tumors (undifferentiated and extraembryonic) and epithelial tumors (cystadenoma, borderline cystadenoma and cystadenocarcinoma) [1, 3]. Cystadenomas are one of the most common ovarian tumors; they arise from the ovarian epithelium and make up for up to 80% of benign ovarian tumors in postmenopausal women [2,4]. Cystadenomas are typically unilateral and have a mean size of 10 cm; in rare scenarios some tumors can grow to giant sizes that exceed 30 cm. Macroscopically, they are smooth tumors containing viscous or serous fluid; on occasions, they can contain small solid areas due to a growth of the fibrous stroma as small papillary formations [1, 2, 4]. They are usually lined by a single layer of epithelial cells without atypia. Regretfully, cystadenomas have been recognized as precursors of ovarian cancer and may slowly transform into borderline tumors and invasive ovarian cancer [1, 5].

Diagnosing a giant abdominal-pelvic mass represents a challenge for the medical team since the clinical examination is usually nonspecific. Imaging studies of the abdomen including CT, ultrasonography or magnetic resonance imaging can aid to distinguish the tumor elements and their features [1, 6, 7]. Although tumor markers, including CA 125 and CAE can be a useful tool for differentiating malignant ovarian tumors, they should not be used in isolation as in some benign tumors these tumor markers can be elevated. Surgery should be the treatment of choice for symptomatic patients or cysts over 10 cm. Some authors advise that fluid aspiration is necessary to reduce operative time and risk-related changes in intra-abdominal pressure. Nonetheless, to reduce oncological and intraperitoneal dissemination, complete extirpation should be completed [1, 3, 5].

Pathology determines if a completion surgery is needed. Nonetheless, there are many issues to contend including the time elapsed from the initial surgery to the final pathology, the waiting time for surgery and the technical and logistical difficulties faced during the completion surgery [8]. Because of this and the fact that this patient was from a remote area with limited access to healthcare, it was decided that with previous authorization from the patient if there would be any doubt during the surgery, the procedure would be radical.

Adnexal masses constitute a challenge especially in resource-limited countries like our own where poor access to health care and noncompliance are usually anticipated. Health care providers should have a broader view of what disease causes in a patient, and all its cultural, economic and social implications. The relationship between the doctors and the patients must be clear at all times to avoid possible misunderstandings especially in cases where patient compliance is limited. Also, in a unique way, this case proves that giant cystadenomas still occur and that prompt and accurate surgical treatment is vital.
